# In Reply to Keskin and Orhan

**DOI:** 10.4274/balkanmedj.galenos.2019.2019.5.43-reply

**Published:** 2019-07-11

**Authors:** Salih Kılıç, Ahmet Çelik, Mehdi Zoghi

**Affiliations:** 1 Clinic of Cardiology, Dr. Ersin Arslan Research and Training Hospital, Gaziantep, Turkey; 2 Department of Cardiology, Mersin University School of Medicine, Mersin, Turkey; 3 Department of Cardiology, Ege University School of Medicine, İzmir, Turkey

To the Editor,

We read the letter that criticized our previous report published in the Balkan Medical Journal. Commentaries to the letter are as noted below (1).

First, in the present subgroup analysis of the WARFARIN-TR study, we aimed to determine inappropriate warfarin and aspirin combination use in patients with warfarin.

Since the guidelines are in line with the inappropriate combination definition for atrial fibrillation patients and patients with warfarin, the main problem is the definition of MHV patients (2,3). For this reason, in our study, we emphasized the differences between the guidelines and determined that the definition of inappropriate combination was according to the ESC guidelines recommendation (4). As the authors determined, the 2017 ESC and the European Association for Cardio-Thoracic Surgery guidelines did not recommend the use of warfarin and aspirin combination in patients with MHV except on specific condition such as thromboembolism despite adequate and inadequate INR (4). In contrast, the ACC/AHA valvular heart disease guideline recommended that aspirin should be routinely added to warfarin therapy in patients with low bleeding risk and MHV (5). These differences between the ESC and ACC/AHA guidelines for MHV patients might result in discrepancies among physicians who are following these guidelines. Although it is not proven by a study, the recommendations of the ESC guidelines are more applied in daily practice in Turkey than those of the ACC/AHA guidelines. Hence, we did our determination in line with the ESC guideline. As a result, we believe that the determination of inappropriate combination is appropriate for our daily practice.

Second, in the method section of our study, we emphasized that because it was not clear in the WARFARIN-TR study how many patients had acute ischemic events or revascularization during the preceding year, we excluded all patients with a history of atherosclerotic disease (ischemic heart disease and peripheral artery disease) or cerebrovascular disease from the subgroup analysis (n=1498; 30.03% of all patients; 1).
